# Gene expression profile analysis of genes in rat hippocampus from antidepressant treated rats using DNA microarray

**DOI:** 10.1186/1471-2202-11-152

**Published:** 2010-11-30

**Authors:** Jun-Ho Lee, Eunjung Ko, Young-Eun Kim, Ji-Young Min, Jian Liu, Yangseok Kim, Minkyu Shin, Moochang Hong, Hyunsu Bae

**Affiliations:** 1 Department of Physiology, College of Oriental Medicine, Kyung Hee University, Hoegi-Dong, Dongdaemun-Ku, Seoul 130-701, Korea

## Abstract

**Background:**

The molecular and biological mechanisms by which many antidepressants function are based on the monoamine depletion hypothesis. However, the entire cascade of mechanisms responsible for the therapeutic effect of antidepressants has not yet been elucidated.

**Results:**

We used a genome-wide microarray system containing 30,000 clones to evaluate total RNA that had been isolated from the brains of treated rats to identify the genes involved in the therapeutic mechanisms of various antidepressants, a tricyclic antidepressant (imipramine). a selective serotonin reuptake inhibitor (fluoxetine), a monoamine oxidase inhibitor (phenelzine) and psychoactive herbal extracts of *Nelumbinis Semen *(NS). To confirm the differential expression of the identified genes, we analyzed the amount of mRNA that was isolated from the hippocampus of rats that had been treated with antidepressants by real-time RT-PCR using primers specific for selected genes of interest. These data demonstrate that antidepressants interfere with the expression of a large array of genes involved in signaling, survival and protein metabolism, suggesting that the therapeutic effect of these antidepressants is very complex. Surprisingly, unlike other antidepressants, we found that the standardized herbal medicine, *Nelumbinis Semen*, is free of factors that can induce neurodegenerative diseases such as caspase 8, α-synuclein, and amyloid precursor protein. In addition, the production of the inflammatory cytokine, IFNγ, was significantly decreased in rat hippocampus in response to treatment with antidepressants, while the inhibitory cytokine, TGFβ, was significantly enhanced.

**Conclusions:**

These results suggest that antidepressants function by regulating neurotransmission as well as suppressing immunoreactivity in the central nervous system.

## Background

Most antidepressants enhance serotonergic or noradrenergic neurotransmission by inhibiting monoamine oxidase or by binding to neurotransmitter transporters, thereby inhibiting neurotransmitter re-uptake. Although antidepressants are widely used to treat depression, they often produce various adverse side effects such as sexual dysfunction, anxiety, blurred vision, headache, sleep disruption, constipation, nausea, sedation, and weight gain [1,2]. There are many classes of antidepressants, including monoamine oxidase inhibitors (MAOIs), tricyclic antidepressants (TCAs), tetracyclic antidepressants (TeCAs), selective serotonin reuptake inhibitors (SSRIs), and serotonin-norepinephrine reuptake inhibitors (SNRIs). Fluoxetine has been shown to be effective for the treatment of depression in long-term controlled trials that also revealed it alleviated anxiety and improved sleep. The tricyclic antidepressant, imipramine, is a dibenzazepine that is primarily used for the treatment of clinical depression and enuresis. Specifically, imipramine is a tertiary amine that inhibits the reuptake of serotonin more effectively than most secondary amine tricyclic antidepressants because it blocks reuptake of the neurotransmitters serotonin and norepinephrine almost equally. Imipramine also exerts activity at the δ-opiate receptors and the dopamine receptors [3]. Phenelzine is a non-selective monoamine oxidase inhibitor (MAOI) that is used as an antidepressant. Phenelzine is a derivative of hydrazine, which is a phenylethylamine-like moiety that is similar to normal substrates of MAO [4,5]. When MAO attempts to oxidize phenelzine, the hydrazine-moiety binds covalently to the enzyme, thereby irreversibly inactivating it. This may contribute to its anxiolytic properties and superior efficacy when treating severe anxiety [6]. Tetracyclic antidepressants (TeCAs) are named after their chemical structure, which contains four rings of atoms. The representative TeCAs, amoxapine and maprotiline, are closely related to tricyclic antidepressants, which contain three rings of atoms. Serotonin-norepinephrine reuptake inhibitors (SNRIs) are used in the treatment of major depression and mood disorders [7]. SNRIs play a role in the inhibition of serotonin and norepinephrine reuptake, which results in an increase in the concentrations of serotonin and norepinephrine and thus an increase in neurotransmission. Most SNRIs, including venlafaxine, desvenlafaxine, and duloxetine, are several fold more selective for serotonin over norepinephrine. SNRIs are sometimes used to treat anxiety disorders, such as obsessive-compulsive disorder (OCD), attention deficit hyperactivity disorder (ADHD), and chronic neuropathic pain [8]. *Nelumbinis Semen *(*NS*) is a traditional medicine that has been used for hundreds of years in East-Asia to treat insomnia, anxiety and women's post-menstrual-pause depression [9]. In previous studies, this herbal medicine was found to exert an antidepressant-like effect in rats when examined by the Porsolt forced swim test. The results of that study also suggested that *Nelumbinis Semen *could increase local cholinergic and dopaminergic or norephinergic neurotransmission via activation of cAMP formation in the hippocampus and pre-frontal cortex [10,11].

The present study was conducted to identify relevant genes that could be involved in the action of four types of antidepressants (fluoxetine, imipramine, phenelzine and *Nelumbinis Semen*). To identify changes in the gene profile after treatment with these four drugs for various lengths of time, we conducted the microarray test for one (acute), three and seven (chronic) days. We also investigated whether genes involved in the acute condition could be replicated in chronic condition or not. The results of this study will likely increase our understanding of the mechanisms by which drug actions occur and provide insight into the way in which cells respond to and adapt to drug exposure.

## Results

### DNA microarray analysis of RNA isolated from pretreated rat hippocampus

**Figure 1:**
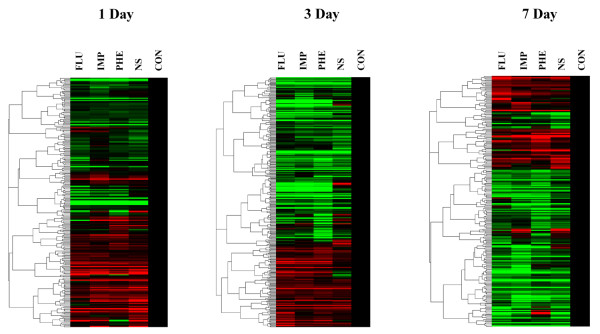
**DNA microarray analysis of RNA isolated from the hippocampus of antidepressant treated rats**. A hierarchical clustergram of genes in treated rat hippocampus was obtained from the control and experimental (antidepressnats) group. Specific pathogen-free, 6 week old male SD rats were divided into four groups that were subjected to the following treatments: fluoxetine, imipramine, phenelzine and *Nelumbinis Semen*. Each group was further subdivided into 3 subgroups (n = 3 per subgroup) that were treated for 1, 3 and 7 days. Fluoxetin (10 mg/kg), imipramine (10 mg/kg) and phenelzine (5 mg/kg) were administrated intraperitoneally, while *Nelumbinis Semen *(400 mg/kg) was administered orally. The control (Con) group was treated with orally administered saline.

We used a genome-wide microarray analysis to examine changes in the gene expression profile of rats after treatment with antidepressants or *Nelumbinis Semen *for one, three and seven days. Of the 30,000 genes evaluated in the present study, the expression of 35, 49, 37 and 44 genes decreased, whereas that of 21, 20, 35 and 27 genes were over-expressed in response to treatment with imipramine, fluoxetine, phenelzine and *Nelumbinis Semen*, respectively (Figure 1). The genes that were significantly repressed and over-expressed in response to treatment are shown in Table 1 and 2.

**Table 1:** List of significantly over-expressed genes affected by antidepressants

Clone name (mRNA)	**Fold change (log**_**2 **_**mean)**				Accession number
	NS	Imip	Fluo	Phen	
Transforming growth factor, beta 2	1.6 (7)	1.8 (7)	2.0 (7)	2.3 (7)	NM_031131
Growth differentiation factor 15	1.3 (7)		1.0 (7)	1.4 (7)	NM_019216
Nerve growth factor, beta	1.0 (3)	1.7 (3)	1.4 (3)	1.7 (3)	XM_001067130
Glial cell line derived neurotrophic factor superfamily receptor alpha 2	1.1 (1)	1.1 (1)	1.0	1.1 (1)	NM_012750
Macrophage expressed gene 1	1.7 (1)	1.4 (1)	1.5 (1)	1.1 (1)	NM_022617
Midkine			1.2 (3)	1.4 (3)	NM_030859
Tumor necrosis factor, member 11		1.4 (1)	1.3 (1)		NM_057149
Type 1 tumor necrosis factor receptor regulator	2.0 (3)	1.7 (3)	1.4 (3)	2.2 (3)	NM_030836
Activity-dependent neuroprotective protein	1.0 (1)		1.3 (1)		NM_012755
Fyn proto-oncogene	1.1 (1)		1.3 (1)	1.1 (1)	NM_012755
Jun D proto-oncogene		2.0 (3)	1.7 (3)	1.9 (3)	NM_138875
RAB6A, member RAS oncogene family		1.1 (7)	1.0 (7)		XM_001062702
Adenosine A1 receptor		1.0 (3)	1.0 (3)	1.0 (3)	NM_017155
Neurotrophic tyrosine kinase, receptor, type 2		1.0 (3)	1.0 (3)		NM_012731
Somatostatin receptor 5		1.5 (3)	1.8 (3)		NM_012882
Leptin receptor	1.1 (3)	1.2 (3)	1.1 (3)	1.5 (3)	NM_012596
Adrenomedullin	1.2 (3)		1.2 (3)	1.1 (3)	NM_012715
Adrenomedullin receptor	1.2 (7)				XM_001076934
Arrestin, beta 1		1.0 (3)	1.5 (3)	1.3 (3)	NM_012910
Regucalcin	1.3 (7)	1.2 (7)		1.9 (7)	NM_031546
Syntaxin 1A (brain)	1.5 (3)			1.3 (3)	NM_053788
Syntaxin 11	1.7 (7)	1.6 (7)	1.7 (7)	2.1 (7)	NM_001025638
Epsin 1	1.2 (3)	1.5 (3)	1.6 (3)	1.4 (3)	NM_057136
Caspase 8		1.5 (3)	1.8 (3)	1.0 (3)	NM_022277
Cyclin-dependent kinase 5 (p35)		1.1 (3)	1.2 (3)	1.1 (3)	NM_053891
Synuclein, alpha		1.0 (3)	1.4 (3)	1.4 (3)	NM_019169
Amyloid beta (A4) precursor protein		1.0 (3)	1.1 (3)	1.3 (3)	NM_019288
Homogentisate 1, 2-dioxygenase		2.4 (7)		2.3 (7)	NM_001012145

List of over-expressed genes affected more than 2 fold to their specific control by antidepressant treatments. ( ) indicated the sacrificed day of oral administered rat with antidepressant. NS; *Nelumbinis Semen*, Imip; imipramine, Fluo; fluoxetine, Phen; phenelzine

**Table 2:** List of significantly repressed genes affected by antidepressants

Clone name (mRNA)	**Fold change (log**_**2 **_**mean)**				Accession number
	NS	Imip	Fluo	Phen	
Immunoglobulin superfamily, member 6	-2.4 (1)		-2.2 (1)	-1.5 (1)	NM_133542
Secreted phosphoprotein 1	-2.3 (1)		-2.5 (1)	-2.2 (1)	NM_012881
Tumor necrosis factor alpha induced protein 6	-1.5 (7)	-1.6 (7)	-1.2 (7)	-1.0 (7)	XM_001065494
Leukocyte cell derived chemotaxin 1	-1.8 (1)	-1.3 (1)			NM_030854
Phospholipase A2, activating protein		-2.5 (1)		-1.6 (1)	NM_053866
Serum amyloid A 4	-1.5 (3)		-1.4 (3)	-1.3 (3)	NM_001009478
Mitogen-activated protein kinase 4		-1.1 (1)			XM_001053505
Tumor necrosis factor receptor superfamily, member 6			-1.8 (1)	-1.1 (1)	NM_139194
Angiopoietin 1	-3.8 (3)		-3.1 (3)		NM_053546
Apoptosis facilitator Bcl-2-like protein 14	-2.5 (3)		-1.0 (3)	-1.0 (3)	NM_001024338
Interferon gamma	-2.4 (3)			-1.0 (3)	NM_138880
Granzyme A		-2.0 (7)	-2.0 (7)	-1.2 (7)	NM_153468

List of repressed genes affected less than 2 fold to their specific control by antidepressant treatments. ( ) indicated the sacrificed day of oral administered rat with antidepressant. NS; *Nelumbinis Semen*, Imip; imipramine, Fluo; fluoxetine, Phen; phenelzine

**Figure 2:**
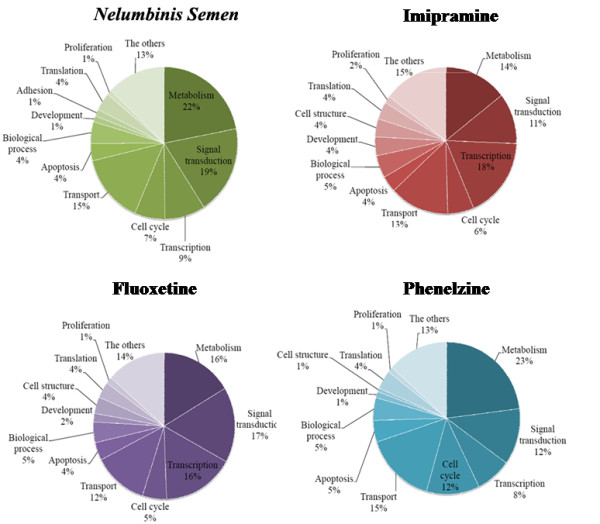
**The functional categories of DEGs identified by DNA microarray analysis of isolated RNA from the rat hippocampus of rats that were treated with antidepressants**. The functional categories of up-regulated genes are shown.

Treatment with *Nelumbinis Semen *resulted in significant up-regulation of four (Glial cell line derived neurotrophic factor superfamily receptor alpha 2, Macrophage expressed gene 1, Activity-dependent neuroprotective protein, Fyn proto-oncogene), six (Nerve growth factor beta, Type 1 tumor necrosis factor receptor regulator, Leptin receptor, Adrenomedullin, Syntaxin 1A, Epsin 1), and five (Transforming growth factor, beta 2, Growth differentiation factor 15, Adrenomedullin receptor, Regucalcin, Syntaxin 11) genes and significant down-regulation of three (Immunoglobulin superfamily member 6, Tumor necrosis factor alpha induced protein 6, Leukocyte cell derived chemotaxin 1), four (Serum amyloid A 4, Angiopoietin 1, Apoptosis facilitator Bcl-2-like protein 14, Interferon gamma), and one (Tumor necrosis factor alpha induced protein 6) genes after one, three and seven days of treatment, respectively. Most of the genes with altered expression were found to be involved with signal transduction, inflammation and cell survival. Treatment with imipramine resulted in significant up-regulation of three (Glial cell line derived neurotrophic factor superfamily receptor alpha 2, Macrophage expressed gene 1, Tumor necrosis factor member 11), 13 (Nerve growth factor beta, tumor necrosis factor receptor regulator, Jun D proto-oncogene, Adenosine A1 receptor, Neurotrophic tyrosine kinase receptor type 2, Somatostatin receptor 5, Leptin receptor, Arrestin beta 1, Epsin 1, Caspase 8, Cyclin-dependent kinase 5, Synuclein alpha, Amyloid beta precursor protein), and five (Transforming growth factor beta 2, RAB6A member RAS oncogene family, Regucalcin, Syntaxin 11, Homogentisate 1, 2-dioxygenase) genes and down-regulation of five (Leukocyte cell derived chemotaxin 1, Phospholipase A2 activating protein, Mitogen-activated protein kinase), one (Interferon gamma), and two (Tumor necrosis factor alpha induced protein 6, Granzyme A) genes after one, three and seven days of treatment, respectively. Treatment with fluoxetine resulted in up-regulation of five (Glial cell line derived neurotrophic factor superfamily receptor alpha 2, Macrophage expressed gene 1, Tumor necrosis factor member 11, Activity-dependent neuroprotective protein, Fyn proto-oncogene), 15 (Nerve growth factor beta, Midkine, Type 1 tumor necrosis factor receptor regulator, Jun D proto-oncogene, Adenosine A1 receptor, Neurotrophic tyrosine kinase, receptor type 2, Somatostatin receptor 5, Leptin receptor, Adrenomedullin, Arrestin beta 1, Epsin 1, Caspase 8, Cyclin-dependent kinase 5, Synuclein alpha, Amyloid beta precursor protein) and four (Transforming growth factor beta 2, Growth differentiation factor 15, RAB6A, Syntaxin 11) genes and downregulation of three (Immunoglobulin superfamily member 6, Secreted phosphoprotein 1, Tumor necrosis factor receptor superfamily member 6), four (Serum amyloid A 4, Angiopoietin 1, Apoptosis facilitator Bcl-2-like protein 14, Interferon gamma) and two (Tumor necrosis factor alpha induced protein 6, Granzyme A ) genes after 1, 3, and 7 days, respectively. Finally, treatment with phenelzine resulted in up-regulation of three (Glial cell line derived neurotrophic factor superfamily receptor alpha 2, Macrophage expressed gene 1, Fyn proto-oncogene), 14 (Nerve growth factor beta, Midkine, Type 1 tumor necrosis factor receptor regulator, Jun D proto-oncogene, Adenosine A1 receptor, Leptin receptor, Adrenomedullin, Arrestin beta 1, Syntaxin 1A, Epsin 1, Caspase 8, Cyclin-dependent kinase 5, Synuclein alpha, Amyloid beta precursor protein), and five (Transforming growth factor beta 2, Growth differentiation factor 15, Regucalcin, Syntaxin 11, Homogentisate 1, 2-dioxygenase) genes and down-regulation of four (Immunoglobulin superfamily member 6, Secreted phosphoprotein 1, Phospholipase A2 activating protein, Tumor necrosis factor receptor superfamily member 6), three (Serum amyloid A 4, Apoptosis facilitator Bcl-2-like protein 14, Interferon gamma), and two (Tumor necrosis factor alpha induced protein 6, Granzyme A) genes after treatment for one, three and seven days, respectively (Table 1 and 2). Taken together, these data demonstrate that antidepressants interfere with the expression of a large array of genes involved in transcription, signaling, survival and protein metabolism (Figure 2).

### Confirmation of the expression of target genes by real time polymerase chain reaction (RT-PCR)

**Figure 3:**
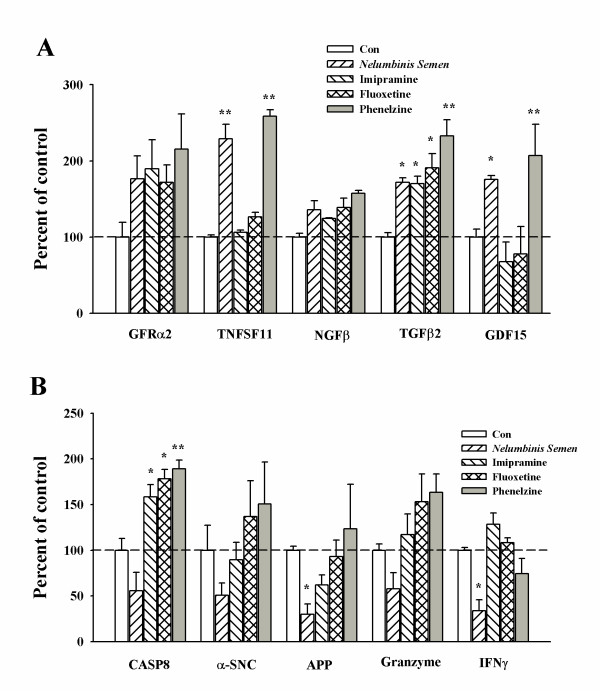
**Quantitative determination of mRNA levels in rat hippocampus obtained from rats that were administered antidepressants as determined by RT-PCR**. Changes in the mRNA levels of DEGs identified in rat hippocampus from rats that had been treated with antidepressants were confirmed by real time PCR. Ten DEGs identified by microarray analysis were selected for confirmation. The dashed line indicates the control mRNA levels. All data were normalized by dividing the results by the amount of GAPDH expressed. Data are presented as mean ± S.E.M. (n = 4) *p < 0.05 and ** p < 0.01 compared to control.

To confirm the differential expression of genes identified by microarray analysis, we analyzed the amount of mRNA that was isolated from the hippocampus of rats that had been treated with antidepressants by real-time RT-PCR using primers specific for selected genes of interest. All of the drugs evaluated in this study induced the up regulation of factors related to cell survival and inflammation, such as GFRα2, TNFSF11, Neff, TGFβ2 and GDF15. However, factors related to neurodegeneration and apoptosis were also up regulated in rats that were treated with imipramine, fluoxetine, and phenelzine. Furthermore, factors related to neurodegenerative diseases, such as CASP8, APP, and SPP1, were not observed in samples obtained from the *Nelumbinis Semen *treated group (Figure 3). Statistical analysis of the data was conducted using the Prism 3.02 software (GraphicPad Software Inc). Error bars represent the mean ± SEM for three samples. * *p *< 0.05, ** *p *< 0.01 compared with control according to ANOVA and Newman-keuls analyses.

## Discussion

In this study, changes in gene regulation profiles in the hippocampus of rats in response to treatment with four different antidepressants, a tricyclic antidepressant (imipramine), a selective serotonin reuptake inhibitor (fluoxetine), a monoamine oxidase inhibitor (phenelzine) and a standardized herbal medicine (*Nelumbinis Semen*), were evaluated by microarray analysis.

Many previous studies of antidepressants have focused on the cAMP cascade and alterations in the synaptic levels of monoamines [12,13]. However, major depression is accompanied by activation of the inflammatory response system, and antidepressants may have immunoregulatory activity. Antidepressants have also been found to exert immunoregulatory effects on human leukocyte functions, especially cytokine production. Furthermore, tricyclic antidepressants and heterocyclic antidepressants can have negative immunoregulatory effects because they inhibit stimulatory cytokines while enhancing inhibitory cytokine production [14,15].

This neuroinflamation and apoptosis includes regulation of intracellular signal transduction pathways and gene expression. Indeed, in this study, treatment with antidepressants was found to primarily be involved with alterations in the expression of several genes related to neuroinflamation and apoptosis. Moreover, the antidepressants evaluated in this study lead to the over-expression of several genes implicated in signal transduction, cell structure, survival, nuclear function and metabolism. Finally, we found that acute administration of antidepressants had an impact on cell cycle and survival, and that chronic treatment resulted in the regulation of factors involved in neuroinflamation and apoptosis. However, the specific gene expression patterns depending on the duration of treatment were not clearly identified in the present study.

### The role of imipramine in gene expression

Treatment with the tricyclic antidepressant, imipramine, resulted in the up regulation of 35 genes and the down regulation of 21 genes involved in signal transduction, metabolism, inflammation, transport, apoptosis, and biological process in general (Table 1 and 2). The DEGs related to cell regulation included TGFβ2 (transforming growth factor, beta 2), NGFβ (nerve growth factor, beta), TNF11 (tumor necrosis factor 11), and RANKL (Receptor Activator for Nuclear Factor κ B Ligand).

Transforming growth factor-beta 2 (TGF-β2) is a cytokine that is known to perform many cellular functions and to play a vital role in embryonic development. TGF-β2 is also known to suppress the effects of interleukin dependent T-cell tumors. RANKL is known as a TNF-related activation-induced cytokine. Overproduction of RANKL is implicated in a variety of degenerative bone diseases, including rheumatism [16]. In addition, RANKL is known to play a role in the immune system, where it is expressed by T helper cells and believed to be involved in cell maturation. Furthermore, RANKL has been shown to be a cell survival factor that is involved in the regulation of T cell-dependent immune response [17].

### The role of fluoxetine in gene expression

Treatment with the selective serotonin reuptake inhibitor, fluoxetine, resulted in the over-expression of 49 genes and the repression of 20 genes (Table 1 and 2); however, the genes affected by fluoxetine varied widely. The affected genes included TGFβ2, JunD (Jun D proto-oncogene) and Adenosine A1 receptor, which were up-regulated, and GZMA (Granzyme A) and IFNγ (interferon gamma), which were down-regulated.

It has been proposed that Jun D proto-oncogene protects cells from p53-dependent senescence and apoptosis. The adenosine A1 receptor is a member of the adenosine receptor group of G protein-coupled receptors that use adenosine as an endogenous ligand. These receptors are believed to take part in the promotion of sleep by inhibiting wake promoting cholinergic neurons in the basal forebrain [18,19]. Granzymes are exogenous serine proteases that are released by cytoplasmic granules within cytotoxic T cells and natural killer cells. Their purpose is to induce apoptosis within virus-infected cells, thereby destroying them [20,21].

### The role of phenelzine in gene expression

Treatment with the monoamine oxidase inhibitor, phenelzine, resulted in increased expression of 35 genes and down-regulation of 37 genes (Table 1 and 2). The genes affected by treatment with phenelzine were involved in various process, and included TGFβ2, GDF15 (growth differentiation factor 15), and LepR (leptin receptor), which were over expressed, and SPP1 (secreted phosphoprotein 1), which was down-regulated.

Growth differentiation factor 15 (GDF15) is a protein that belongs to the transforming growth factor beta superfamily and plays a role in the regulation of inflammatory and apoptotic pathways activated in response to tissue injury and during disease processes. Furthermore, the expression of GDF15 in liver can be significantly up-regulated in response to injury of the liver, brain, kidney, heart and lung [22,23]. Leptin, which is produced by adipose tissue, plays a key role in the regulation of energy intake and expenditure, including the regulation of appetite. Specifically, leptin binds to the ventromedial nucleus of the hypothalamus, which sends a signal to the brain indicating that the body has had enough to eat [24]. Secreted phosphoprotein 1 (SSP1) is an extracellular structural protein and an organic component of bone. High levels of SSP1 expression are correlated with tumor invasion, progression or metastasis in multiple forms of cancer. SSP1 mediates the molecular mechanisms that determine metastatic spread, such as the prevention of apoptosis, extracellular matrix proteolysis, remodeling and cell migration [25].

### The role of *Nelumbinis Semen *in gene expression

*Nelumbinis Semen *has been widely used in traditional medicine as a remedy for insomnia, anxiety and women's depression following menopause. In previous reports, we found that this herbal medicine produced an antidepressant effect on rats under a forced swim-induced depression-like symptom [11], as well as a chronic mild stress (CMS)-induced depression-like symptom [10]. We also recently conducted a study that suggested that *Nelumbinis Semen *increases serotonin levels, which are normally decreased in depression, thereby enhancing central serotonergic transmission and possibly providing therapeutic action for the treatment of depression. This is similar to the results of other studies that have suggested that *Nelumbinis Semen *induces an antidepressant effect through the enhancement of serotonin [9].

Treatment with *Nelumbinis Semen *resulted in increased expression of 44 genes and decreased expression of 27 genes (Table 1 and 2). The genes affected by *Nelumbinis Semen *were involved in various fields and included TGFβ2 and GFRα2 (glial cell line derived neurotrophic factor family receptor alpha 2), which were up-regulated, as well as IFNγ, which was downregulated.

Glial cell derived neurotrophic factor, also known as GDNF, is a small protein that promotes the survival of a wide variety of neurons [26]. This gene encodes a highly conserved neurotrophic factor. In addition, the recombinant form of this protein is known to promote the survival and differentiation of dopaminergic neurons in culture, and to prevent axotomy induced apoptosis in motor neurons [27,28]. IFNγ (also known as immune interferon) is a dimerized soluble cytokine that is the only member of the type II class of interferons and is secreted by T lymphocytes [29]. IFN-γ has antiviral, immunoregulatory, and anti-tumor properties [30].

The molecular and biological mechanisms by which antidepressant action occurs are not fully understood, and various explanations of the expression profiles generated in many similar studies have been offered. In this study, we found that DNA microarrays provided an efficient tool for evaluating changes in gene expression in response to treatment with antidepressants. The use of this method allowed us to isolate several mRNAs from the rat hippocampus that were differentially expressed in response to treatment with antidepressants. Some of these genes have been identified in previous studies and others are candidates for further study. In the present study, administration of three antidepressants and a traditional herbal medicine was found to affect a wide range of genes involved in various processes. For example, all four drugs induced the up regulation of factors related to cell survival such as TGFβ, NGFβ, GFRα2, and TNF. These results suggest that antidepressants regulate the cAMP cascade and alterations in synaptic levels of monoamines, as well as cell survival and neuroinflamation. However, some factors associated with neurodegenerative diseases were up-regulated in response to treatment with the three antidepressants evaluated in this study (imipramine, fluoxetine, and phenelzine) (Table 1 and 2). These factors included caspase 8, α-synuclein, amyloid β precursor protein and cyclin-dependent kinase. Caspase 8 plays a central role in the execution-phase of cell apoptosis. Synuclein selectively inhibits phospholipase D2 and is abundant in neurofibrillary lesions of patients with Alzheimer's disease [31]. Dysregulation of cyclin-dependent kinase 5 (CDK5) has been implicated in several neurodegenerative diseases, including Alzheimer's [32,33]. Taken together, these results show that the three commonly used medications evaluated in this study have the potential to produce adverse effects. However, *Nelumbinis Semen *did not up-regulate any factors related to neurodegenerative diseases.

Major depression is associated with an increased secretion of IFNγ. Moreover, the administration of IFNγ results in behavioral effects and depression-like syndromes [34,35]. Antidepressants have been found to significantly enhance the production of another inhibitory cytokine, TGFγ. This cytokine is known to inhibit several immune reactions, including autoimmune disorders [14]. Interestingly, we found a general trend in which production of the stimulatory cytokine, IFNγ, was decreased in response to treatment with all of the antidepressants evaluated in this study, while production of the inhibitory cytokine, TGFγ, was significantly enhanced by treatment with the antidepressants evaluated here.

Many previous studies have reported that antidepressant-induced alterations in mRNA expression are involved in stress and survival, the cell cycle, neurotransmission, and metabolism in general [36-38]. When combined with our findings, these observations suggest that antidepressants with different molecular structures share similar targets at the molecular level.

## Conclusion

Our results demonstrate that antidepressants interfere with the expression of a large array of genes involved in various processes, suggesting that the therapeutic effect of these antidepressants is very complex. Unlike other antidepressants, we found that the standardized herbal medicine, *Nelumbinis Semen*, is free of factors that can induce neurodegenerative diseases.

## Methods

### Animal and drug treatment

This study was conducted using specific pathogen-free, six week old male Sprague-Dawley (SD) rats (Charles River Technology Inc. Korea). The SD rats were housed in a controlled environment (12:12-hour light:dark cycle, temperature 23 ± 2°C, humidity 50 ± 10%) with water and food available *ad libitum*. The rats were divided into four groups that were subjected to the following treatments: fluoxetin, imipramine, phenelzine and *Nelumbinis Semen*. The three antidepressants were dissolved in saline while the *Nelumbinis Semen *was dissolved in distilled water. Each group was further subdivided into three subgroups (n = 3 per subgroup) that were treated for 1, 3 and 7 days with one of the four drugs. Fluoxetin (10 mg/kg), imipramine (10 mg/kg) and phenelzine (5 mg/kg) were administrated intraperitoneally, while *Nelumbinis Semen *(400 mg/kg) was administered orally by intubation using a stainless steel ball-tipped gavage needle attached to an appropriate syringe. The day after treatment, the rats were sacrificed and their hippocampuses were collected by dissection from removed each hemisphere. The samples were then immediately frozen with liquid nitrogen and stored at -80°C until RNA isolation. There was also a control group of rats that were only treated with saline. Fluoxetin, imipramine and phenelzine were obtained from Sigma (St. Louis, MO, USA), and *Nelumbinis Semen *was purchased from Sun Ten Pharmaceutical (Taipei, Taiwan). The experiments began when the rats were six weeks old, and all of the procedures involving animals were approved by the Institutional Animal Care and Use Committee of Kyung Hee University.

### RNA preparation

RNA was extracted from the rat hippocampus using an Rneasy^® ^mini kit (Qiagen GmbH, Hilden, Germany) according to the manufacturer's instructions. The extracted RNA was then quantified using NanoDrop (NanoDrop Technologies, Inc ND-1000; Wilmington, DE, U.S.A).

### Oligonucleotide chip microarray

An oligonucleotide chip microarray was performed using single round RNA amplification protocols, following the Affimetrix specifications (Affimetrix GeneChip Expression Analysis Technical Manual). Briefly, 3 micrograms of total RNA were used to synthesize first-strand complementary DNA (cDNA) using oligonucleotide probes with 24 oligo-dT plus T7 promoter as primers (Proligo LLC, Boulder, CO, USA) and the Superscript Choice System (Life Technologies, Invitrogen, Milan, Italy). After double-stranded cDNA synthesis, the products were purified by phenol-chloroform extraction, and then biotinylated antisense complimentary RNA (cRNA) was generated through in vitro transcription using a Genechip Expression 3'-Ampliflcation Reagent for IVT Labeling kit (Affymetrix, Inc. USA). Next, the biotinylated labeled cRNA was fragmented, and 10 μg of the total fragmented cRNA was then hybridized to the Affymetrix Rat 230 2.0 GeneChip array (P/N900470, Affymetrix Inc., USA). The Affimetrix Fluidics Station 450 was then used to wash and stain the chips, after which the nonhybridized target was removed. Next, the samples were incubated with a streptavidin-phycoerythrin conjugate to stain the biotinylated cRNA. The staining was then amplified using goat IgG as a blocking reagent and biotinylated antistreptavidin antibody (goat), followed by a second staining step using a streptavidin-phycoerythrin conjugate. The fluorescence was detected using the Genechip System Confocal Scanner (Hewlett-Packard), and analysis of the data contained on each GeneChip was conducted using the GeneChip 3.1 software produced by Affymetrix, using the standard default settings. To compare different chips, global scaling was used, with all probe sets being scaled to a user-defined target intensity of 150. Total of 45 rats (5 group × 3 subgroup × 3 individual) were separately performed microarray analysis.

### Data analysis

The MAS5 algorithm was used to evaluate the expression signals generated by the Affymetrix Rat 230 2.0 array. Global scaling normalization was then performed, after which the normalized data were log-transformed using base 2. Next, fold change and a Welch t-test were used to select the differentially expressed genes (DEGs) using a fold change threshold of 2.0-fold and a P < 0.05 to indicate significance. Each probe set used in the Affymetrix GeneChip produces a detection call, with P (present call) indicating good quality, M (marginal call) indicating intermediate quality and A (absent call) indicating relatively low reliability. Therefore, probe sets that resulted in A calls in the compared groups were removed to avoid false positives. A volcano plot was used to better visualize and compare the two DEG methods. The 2.0-fold DEGs were clustered using the GenPlex™ v2.3 software (ISTECH Inc., Korea) using hierarchical clustering with Pearson's correlation as a similarity measure and complete linkage as the linkage method. In addition, gene ontology significance analysis was conducted to evaluate the functional relationships among the 2.0-fold DEGs using high-throughput GoMiner. The 2.0-fold DEGs were then mapped to the relevant pathways using the GenPlex™ v2.4 software (ISTECH Inc., Korea). The pathway resources were provided by the KEGG database.

### Real-time RT-PCR analysis

Microarray verification was performed by real-time RT-PCR analysis of selected genes using SYBR Green I Master Mix (Applied Biosystems, Foster City, CA, U.S.A) and primers (Genotech Inc. Korea). Complementary DNA (cDNA) was synthesized using 1 μg of total RNA in a reverse transcription reaction. Real time-PCR quantitative mRNA analyses were performed with an Applied Biosystems 7300 Real Time PCR System using the SYBR Green fluorescence quantification system (Applied Biosystems, Foster City, CA, U.S.A) to quantify the amplicons. The PCR conditions were 40 cycles of 95°C (15 sec), 60°C (1 min), and a standard denaturation curve. PCR conditions for each target were optimized according to the primer concentration, the absence of primer dimmer formation, and the efficiency of amplification of both the target genes and the housekeeping gene control. PCR reactions were carried out in a total volume of 20 μL in PCR master mix containing 10 μL 2X SYBR Green, 5 μM each of sense and antisense primer, and 2 μL of 1:2 diluted cDNA filled up to 20 μL with DEPC-treated H_2_O. To normalize the cDNA content of the samples, we used the comparative threshold (C_T_) cycle method, which consists of the normalization of the number of target gene copies versus the endogenous reference gene, GAPDH. The C_T _is defined as the fractional cycle number at which the fluorescence generated by cleavage of the probe passes a fixed threshold baseline when amplification of the PCR product is first detected.

Statistical analysis of the data was carried out using the Prism 3.02 software (GraphicPad Software Inc). Error bars represent the mean ± SEM for three samples. * *p *< 0.05, ** *p *< 0.01 compared with control according to ANOVA and Newman-keuls analyses.

## Authors' contributions

JHL participated in the design of the study and in drafting and finalizing the manuscript. EK, YEK, JL and JYM carried out the animal care, microarray and RT-PCR analysis, respectively. YK, MS, MH and HB participated in the design of the study. All authors read and approved the final manuscript.
